# Current and emerging therapies for knee osteoarthritis: From conventional approaches to machine learning and stem cell innovations

**DOI:** 10.1016/j.reth.2026.101106

**Published:** 2026-04-26

**Authors:** Arthur W. Cowman, Jordan N. Tang, Jeffrey Deng, Ammar Abu-Halawa, Haiyue Jin, Christopher Z. Liu, Ryan Hoang, Walter Cowman, Michael S. Kim, Sultan Baz, MacKinnly T. Knoerzer, Amir-Ala Mahmoud, Amir-Ali Mahmoud, Miles D. Melamed, Hong-wen Deng

**Affiliations:** a University of California Irvine School of Medicine, Irvine, CA, 92617, USA; b Geisel School of Medicine, Dartmouth College, Hanover, NH, 03755, USA; c Department of Plastic Surgery, University of California Davis, Sacramento, CA, 95817, USA; d Department of Radiology, University of California Davis, Sacramento, CA, 95817, USA; e University of California San Diego School of Medicine, San Diego, CA, 92093, USA; f Tulane Center for Biomedical Informatics and Genomics, Deming Department of Medicine, School of Medicine, Tulane University, New Orleans, LA 70112, USA

**Keywords:** Knee osteoarthritis, Mesenchymal stem cells, Total knee arthroplasty, Cartilage regeneration, Machine learning

## Abstract

Osteoarthritis is characterized by cartilage degradation and joint distortion. The most prevalent version of osteoarthritis, knee osteoarthritis (KOA), affects millions, acting as a leading cause of disability. With the average life expectancy continuing to grow, the global burden of KOA is expected to increase. KOA involves chronic pain that limits function and adversely impacts patients’ quality of life. Long-term management is costly and burdensome, often requiring continuous treatment that only delays disease progression unless patients receive a total knee arthroplasty. Recently, traditional treatment modalities for KOA have been augmented by advancements in machine learning (ML), which enable automated disease grading, predictive modeling for early diagnosis, and personalized treatment strategies, alongside growing interest in stem cell-based therapies. With the goal of developing less invasive treatments that slow or reverse KOA progression, investigation into these novel therapies have increased. Stem cell-based therapies offer the potential to modulate inflammation, reduce pain, and preserve cartilage integrity, addressing key limitations of existing treatments. Mesenchymal stem cells have shown promising preclinical and clinical results, demonstrating improvements in pain, function, and cartilage-related outcomes through immunomodulatory and paracrine mechanisms. This narrative review synthesizes current evidence on conventional and emerging therapies for KOA, with a dual focus on ML-driven clinical applications and stem cell-based regenerative strategies, and discusses their potential roles in improving diagnosis, treatment selection, and patient outcomes. While further research is needed to understand the full potential of stem cells for treating KOA, these promising findings make it a useful topic for current and future clinical applications.

## 1 Introduction

Globally, knee osteoarthritis (KOA) is the 11th leading cause of disability, characterized as a chronic degenerative joint disease with clinical symptoms and joint tissue distortion [1]. The pain associated with KOA is the most common reason for activity limitations in adults, resulting in a sedentary lifestyle and comorbidities such as cardiovascular disease that contribute to a 20% higher age-adjusted mortality [[2], [3], [4]]. In 2013 alone, more than 300 billion dollars were spent on medical care costs and lost wages due to osteoarthritis (OA) [5]. As the life expectancy of patients continues to grow, the prevalence of KOA is expected to drastically increase [[3], [4], [5], [6], [7], [8]]. With an estimated 22.9% of the global population of patients 40 years and older living with KOA, average life expectancy increasing, and no cure in sight, it is crucial to address the limitations of KOA treatment to prevent further global burden and suffering [6,7].

Primary treatments for KOA include physiotherapy, nonsteroidal anti-inflammatory drugs (NSAIDs), pain-relieving agents, hyaluronic acid (HA), and corticosteroid (CS) injections. Unfortunately, none of these treatments reverse the progressive deterioration of the knee joint, and if symptoms become severe enough, surgical interventions may become necessary [9,10]. Consequently, there is great interest in novel non-surgical therapies aimed at alleviating the symptoms of KOA [11].

Mesenchymal stem cells (MSCs) have emerged as a potential therapy due to their ability to self-renew, exhibit multilineage differentiation, and be easily harvested from a variety of tissues [12]. Numerous studies have already indicated the usefulness of MSCs in alleviating pain, improving function, and influencing knee cartilage volume [3,10,[13], [14], [15]]. Stem cells may solve the challenging problem of reversing the degenerative properties of KOA [16]. In parallel, recent advancements in machine learning (ML) have introduced powerful tools for improving diagnostic precision, disease severity classification, and treatment outcome prediction in KOA. By leveraging large datasets of clinical and imaging data, ML algorithms can detect subtle radiographic changes, identify patient-specific risk factors, and aid in developing personalized management strategies.

To utilize these novel therapies, it is imperative that physicians acquire a basic understanding of KOA, ML, and advances in stem cells. This review aims to provide a comprehensive overview of KOA pathophysiology, current and emerging treatment modalities along with their limitations, and the therapeutic potential of ML and stem cells in the context of KOA.

## 2 Anatomy and pathophysiology of knee osteoarthritis

### 2.1 Knee joint anatomy and cartilage composition

As the largest synovial joint within the human body, the knee contains many important anatomical structures, including bones, cartilage, ligaments, infrapatellar fat pad, and synovium [17]. Given that KOA involves extensive cartilage breakdown, it is important to understand the role of hyaline cartilage and fibrocartilage as well as the mechanisms by which cartilage deteriorates.

Hyaline cartilage serves as a smooth, frictionless surface that permits movement of bones and absorption of mechanical shock [18]. In the knee, hyaline cartilage that lines the surface of each bone within the synovial joint is specifically known as articular cartilage [15]. This form of cartilage is composed of mostly water (>70%) and has chondrocytes (2-3% of cartilage volume) located within the organic extracellular matrix, which mainly consists of type II collagen, aggrecan, and other proteoglycans [15,19]. Moreover, within the knee, there are two crescent-shaped pieces of cartilage with wedge-like cross sections located between the femoral condyles and the tibial plateau. These two fibrocartilage-containing structures are referred to as the menisci. Compared to hyaline cartilage, fibrocartilage contains fewer chondrocytes and proteoglycans but more type I and type II collagen. This configuration creates a densely interwoven collagen fibril network, which allows for greater resistance to compression. The composition, location, and shape contribute to the menisci's ability to function as a shock absorber and a secondary stabilizer of the knee joint [[20], [21], [22]].

### 2.2 Cartilage homeostasis and molecular pathogenesis of KOA

**Fig. 1:**
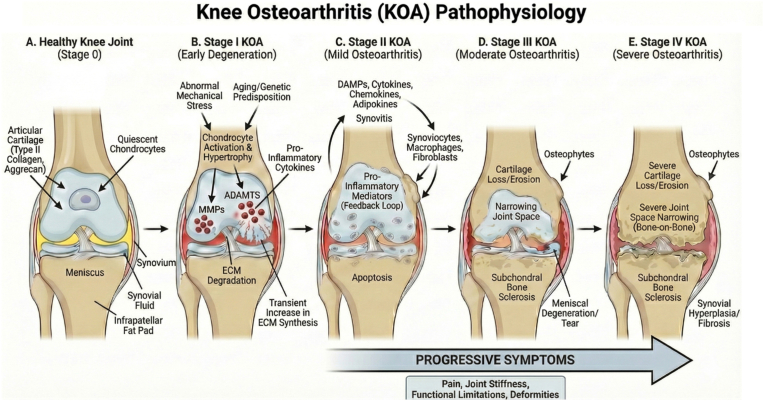
**Pathophysiological Progression of Staged Knee Osteoarthritis (KOA).** This schematic illustrates the structural and molecular mechanisms driving KOA progression from a healthy knee joint (Stage 0) to severe osteoarthritis (Stage IV). **(A**–**B)** Initiating factors, such as abnormal mechanical stress and aging, trigger chondrocyte activation and the release of catabolic enzymes (MMPs, ADAMTS) and pro-inflammatory cytokines, causing early extracellular matrix (ECM) degradation [28]. **(C)** A vicious inflammatory loop involving damage-associated molecular patterns (DAMPs), activated synoviocytes, and macrophages leads to synovitis and further chondrocyte apoptosis. **(D**–**E)** Continued degeneration results in severe cartilage erosion, significant joint space narrowing leading to a “bone-on-bone” state, osteophyte formation, subchondral bone sclerosis, and synovial fibrosis. This progressive structural damage correlates with worsening clinical symptoms, including chronic pain, stiffness, functional limitations, and deformities [26,29,30].

The etiology of KOA is multifactorial, including a complex interplay of aging, malalignment, obesity, trauma, prior surgeries, and genetic predispositions [23]. Disease progression usually takes many years and is accompanied by worsening symptoms [17]. Early stages begin with the alteration of the extracellular matrix surrounding chondrocytes [24]. In homeostasis, chondrocytes are quiescent within the joint, expressing minimal metabolic activity [16,23]. However, when chondrocytes detect mechanical stressors, such as trauma or abnormal mechanical forces, they endure a transient increase in metabolism and proliferation. The production of type II cartilage, aggrecans, and other extracellular matrix proteins is upregulated to initiate repair of damaged extracellular matrix [16,23,25]. Concurrently, these hypertrophic chondrocytes express collagen type X, which marks the terminal differentiation of chondrocytes that modulate the expression of zinc-dependent metalloproteinases (MMPs), as well as A Disintegrin and Metalloproteinase with Thrombospondin motifs (ADAMTS) [17,26]. MMPs and ADAMTSs are the primary cartilage extracellular matrix-degrading enzymes [27]. Although chondrocytes recognize damage and attempt to restore cartilage, the proteolytic activity of MMPs and ADAMTSs degrades the proteoglycan and collagen network of cartilage faster than the rate at which chondrocytes can synthesize components of the extracellular matrix. Eventually, the catabolic effect of metalloproteinases leads to a total loss of cartilaginous tissue within the joint (Fig. 1) [26].

### 2.3 Structural joint degeneration and clinical consequences

The elimination of articular cartilage results in numerous structural changes. Osteoblasts build up subchondral bone, resulting in osteophytes, the joint space between the femur and tibia narrows, and subchondral sclerosis occurs [20]. Without cartilage, friction between bones results in intense pain, limited mobility, and weakening of muscles and tendons (Fig. 1) [23]. Unfortunately, without a better understanding of the molecular mechanisms that contribute to cartilage degradation and advancement of KOA, we are limited in the therapeutic approaches we can take to overcome the body's inability to regenerate lost cartilage [23,26].

### 2.4 Implications for therapeutic targeting

The progressive failure of cartilage homeostasis in KOA is the result of an imbalance between catabolic enzymatic degradation and its counterregulatory anabolic repair mechanisms. Chondrocyte hypertrophy, metalloproteinase activity, and subchondral bone remodeling collectively contribute to irreversible structural and functional cartilage loss. The avascular nature of articular cartilage severely limit endogenous repair capacity, which is the main factor in why modern treatment of KOA remains largely palliative. These pathophysiologic constraints underlie the biologic rationale for current therapeutic strategies, which aim to slow the inflammatory cascade, preserve extracellular matrix integrity, and augment intrinsic chondrogenic repair. Likewise, research into both novel cell-based therapies and data-driven approaches follow the same principles.

## 3 Traditional approaches to knee osteoarthritis

The type of treatment modality used by physicians is dependent upon the nature and severity of patients’ KOA, medical history, and risk factors. The progressive disease impairs the entire joint and atrophies surrounding muscles, with long-term sequelae often including a decrease in physical activity, impaired sleep, disability, depression, and adaptive pain processing in the peripheral or central nervous systems [8]. Thus, physicians utilize an algorithmic multimodal approach that starts with nonpharmacologic and pharmacological therapies, invasive procedures, and ultimately ends with surgery.

### 3.1 Non-pharmacological therapies

Recent studies evaluating the efficacy of nonpharmacologic therapies highlight their proactive approach regarding their ability to potentially provide long-term relief and delay and even prevent or alter disease progression. In patients with KOA, land-based exercise has been shown to decrease pain and increase function, with continued benefits lasting between 2 and 6 months following treatment [31]. Furthermore, Quicke et al. found that another systematic review of low-impact exercise in adult patients with KOA revealed zero increase in both subjective pain reporting and objective imaging analysis. Intentional exercise programs designed to enhance muscle strength, neuromuscular control, balance, and kinesthetic awareness may also reduce the risk of falls and subsequent inflammatory cascades in already vulnerable joints [32]. Provided obesity is a strong risk factor for KOA, weight loss through exercise and diet programs has been investigated to alleviate the mechanical stress on the joint and promote health. Studies have noted that, with overweight patients, a combined program of exercise and diet resulted in more weight loss, reduced pain, and increased functionality—demonstrating a synergistic effect compared to either therapy alone [33]. However, diet may only play an additive effect in the presence of exercise. For instance, Hall et al. reported a moderate decrease in pain with a combined program, but not with diet itself [34]. The long-term benefits of weight loss in an observational study of overweight or obese patients resulted in diminished rates of knee cartilage degeneration as assessed by MRI [35].

### 3.2 NSAIDS

**Fig. 2:**
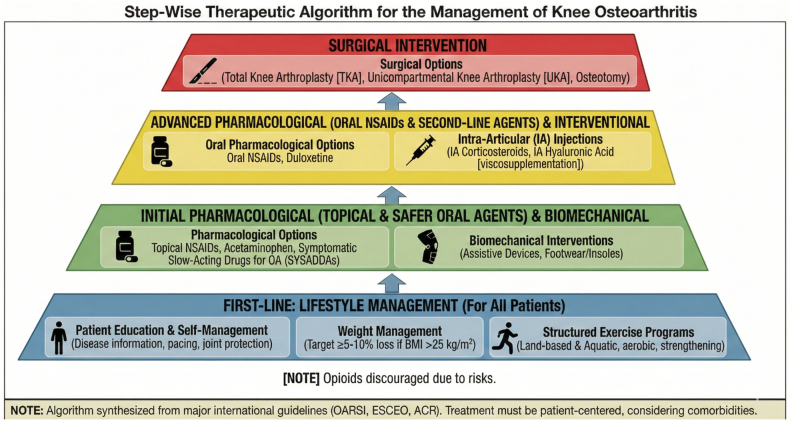
**Conventional Step-Wise Therapeutic Algorithm for Knee Osteoarthritis.** This figure illustrates a tiered, stepped-care approach to knee osteoarthritis (KOA) management, synthesizing recommendations from major international consensus guidelines (e.g., OARSI, ESCEO, ACR). The foundational base layer (lifestyle management, education, and exercise) is intended for implementation across all patient profiles. Progression to subsequent, more advanced pharmacological and interventional tiers (indicated by the upward arrows) is recommended only when symptomatic response to initial conservative measures is inadequate. Surgical intervention is reserved as a last resort for severe, refractory disease. Clinical decision-making at every stage must be patient-centered, heavily weighing individual comorbidities and safety profiles [37,40].

For most patients suffering from KOA, topical NSAIDs are the first pharmacologic treatment approach. Topical NSAIDs are applied directly to the affected sites and work by permeating the skin to inhibit cyclooxygenase enzymes (COX) responsible for inflammation [36]. This delivery system provides a local analgesic and anti-inflammatory effect while avoiding the complications associated with oral intake of NSAIDs [37]. However, when topical NSAIDs are ineffective or multiple joints are involved, oral NSAIDs are suggested if patients do not have contraindications (Fig. 2) [37,38]. Osteoarthritis Research Society International (OARSI) guidelines support a combination of nonselective NSAIDs with proton-pump inhibitors or a COX-2 inhibitor for patients with gastrointestinal comorbidities [37]. Oral NSAIDs are contraindicated for patients with cardiovascular comorbidities since a meta-analysis of close to 500,000 patients demonstrated an association between NSAID use and an increased risk of myocardial infarction [39]. In addition, patients with gastrointestinal issues may be at a higher risk of developing stomach ulcers and are thus not advised to take oral NSAIDs. Topical and oral NSAIDs can be used to manage the pain associated with KOA but do not ameliorate existing joint damage or halt disease progression while adding potential side effects in patients with gastrointestinal and cardiovascular conditions.

### 3.3 Intra-articular injections

CS injections, such as triamcinolone and methylprednisolone acetate, have been extensively used by clinicians as anti-inflammatory agents that suppress inflammation within the affected knee joint [41]. Despite this, a Cochrane review reported the same pattern observed in numerous randomized clinical trials (RCTs) after CS injection: low-moderate initial pain improvement, steady decline in pain reduction, and zero lasting effect around 26 weeks (about 6 months) post-injection [42]. Furthermore, repeated injections are not suggested, as injections of CS injections may result in cartilage and soft tissue degeneration [43]. Though it may be useful to quell inflammation flare-ups or as a prophylactic in anticipation of future strenuous activity, CS injections should not be used as a long-term treatment for KOA.

As a major component of synovial fluid and articular cartilage, HA lubricates the knee joint, relieving pain, and polymerizes with proteoglycan to halt its precipitation from the cartilaginous matrix—significantly alleviating symptoms of KOA in some patients [[44], [45], [46]]. The mechanism of action of HA injection therapy is not fully understood but is thought to increase shock absorption, increase elasticity, and decrease friction in the knee joint [47]. A review of 14 meta-analyses lacked sufficient quality evidence to evaluate HA injections versus placebo; however, HA injections showed similar efficacy to oral NSAIDs with fewer side effects [48]. HA injections demonstrated a longer duration of pain reduction (26 weeks) compared to CS injections (3-4 weeks) [49]. In contrast to CS injections, repeated HA injections seemed to be safe and efficacious up to 25 months—with the most common side effects being joint swelling and arthralgia [46]. Since knees affected by OA have up to 50% less HA than healthy knees due to reduced synthesis, accelerated breakdown, and higher clearance, HA injections may alter the progression of KOA, though larger RCTs and more evidence are needed [50,51].

Platelet-rich plasma (PRP) has recently gained enthusiasm in the treatment of KOA due to the high concentration of growth factors present in its composition, including platelet-derived growth factor, insulin-derived growth factor, epidermal growth factor, and vascular endothelial growth factor, among others. These biological factors in supraphysiologic concentrations are hypothesized to change the intra-articular microenvironment to promote chondrocyte proliferation and angiogenesis and reduce inflammation, though the exact mechanism is still unclear [52,53]. PRP is autologously derived from blood, centrifuged, and activated with calcium solution prior to administration into the knee. However, there is currently no standardization between preparation protocols, which makes it difficult to assess and draw conclusions on its efficacy in the treatment of KOA across studies [53]. A double-blind clinical trial comparing PRP and normal saline placebo showed no significant difference in primary outcomes in the treatment of KOA, measured as change of symptoms after 12 months and percentage change in MRI measured medial tibial cartilage volume after 12 months [54]. However, other studies found statistically significant differences between PRP and normal saline pain scores at 12 months [55]. Interestingly, they also found a statistically significant decrease in pain scores between groups receiving 1 or 3 intra-articular PRP injections, indicating a benefit of repeating PRP injections every 6 months. There has also been interest in leukocyte-rich platelet-rich plasma (LRPRP) and leukocyte-poor platelet-rich plasma (LPPRP) in the treatment of KOA. One study analyzing the difference between LPPRP and LRPRP showed a statistically significant improvement in both groups as compared to baseline, but no statistically significant difference between the groups at 12-month follow-up [56]. The presence of leukocytes in PRP is thought to produce a pro-inflammatory environment, though the role this plays in the treatment of KOA with PRP is still under debate [52,56,57].

### 3.4 Surgical interventions

For patients with severe KOA, who are resistant to conservative therapy with pain that is significantly adversely affecting their quality of life, surgical interventions, including high tibial osteotomy (HTO) and TKA are often performed (Fig. 2).

The goal of HTO is to realign the leg and shift weight-bearing load from a damaged area to a healthier area. Although HTOs have recently risen in popularity for KOA, around 4-26% of patients do not report satisfactory pain relief, and many opt for revision to TKA [[58], [59], [60]]. Furthermore, for optimal outcomes, the selection of an ideal candidate for HTO includes young age (<60 years), no existing articular damage (Ahlback grade III or more), isolated medial OA, and good range of motion [61].

TKA is the final surgical option for treating KOA and is the accepted treatment when substantial quantities of cartilage have been degraded in end-stage disease and clinical symptoms are too severe to manage with less invasive modalities. Surgery entails replacing the entire knee joint with a prosthesis. Most patients report improved quality of life, physical function improvements, and pain reduction following this operation. However, it is not unusual for patients to require revision surgeries [62]. Although this procedure does provide substantial relief and restoration of knee functionality, TKA serves as a last resort that is expensive and invasive [63]. The procedure comes with numerous potential postoperative complications, such as blood clots, infections, and misalignment of the metal implants. Furthermore, the literature demonstrates that nearly 30% of patients who received a TKA are still dissatisfied with their overall quality of life after surgery [64,65]. With over 3 million TKA surgeries predicted by the year 2030, it is increasingly important that a new, less expensive and invasive form of regenerative therapy be developed [62]. Continued exploration of novel therapeutic approaches for KOA is imperative to proactively prevent, cease, or stimulate regeneration of the cartilage deterioration that arises during KOA progression.

## 4 Machine learning approaches in knee osteoarthritis management

### 4.1 Machine learning-based diagnosis and severity assessment

Management of KOA benefits from accurate diagnosis and severity assessment. ML technologies have significantly advanced the diagnosis and severity assessment of KOA, transforming traditional imaging methods through automation and enhanced accuracy. Convolutional Neural Networks (CNNs) are a standout technology in this domain, with their capability to analyze complex patterns in medical images. Studies have demonstrated the efficacy of CNNs in identifying KOA severity by processing large datasets of knee radiographs, which allows for the detection of features that might be overlooked by human observers. For example, the application of CNNs in grading KOA according to the Kellgren-Lawrence (KL) scale has been shown to improve consistency and accuracy, providing a more objective standard for diagnosing the disease [66,67]. This automation not only aids radiologists by reducing their workload but also increases diagnostic throughput, facilitating earlier and more precise interventions.

In addition to CNNs, other ML models, such as decision trees and support vector machines, have been applied to radiographic data to classify KOA severity. These models leverage pattern recognition capabilities to differentiate between normal and osteoarthritic knee joints based on characteristic changes such as joint space narrowing and osteophyte formation. By integrating clinical data, such as patient history and demographic information, these models enhance their predictive accuracy, allowing for a more comprehensive assessment of KOA severity and informing management of complex cases. Moreover, the use of explainable AI methods provides insights into the decision-making process of these models, ensuring that their outputs are interpretable and clinically relevant​ [68].

Even with these advancements, challenges remain in the widespread adoption of ML models for KOA diagnosis. The performance of these models heavily relies on the quality and diversity of training datasets, which must represent a wide range of patient populations and disease manifestations [68]. There is also a need for rigorous external validation to ensure the generalizability of these models across different clinical settings. Furthermore, addressing ethical and regulatory considerations, such as data privacy and algorithmic bias, is essential to foster trust and acceptance among healthcare providers and patients [67].

### 4.2 Risk stratification and disease progression modeling

ML is transforming the identification of risk factors associated with the development and progression of KOA, offering new insights into disease etiology and potential preventive strategies. By analyzing large, multifaceted datasets, ML models can uncover complex interactions between genetic, demographic, and environmental factors that contribute to KOA risk [68,69]. For instance, unsupervised learning techniques, such as clustering algorithms, have been used to categorize patients into subgroups based on shared characteristics, enabling the identification of unique risk profiles and facilitating targeted prevention efforts. These insights can inform clinical decision-making, allowing for earlier interventions and personalized management plans to mitigate disease progression.

In addition to identifying traditional risk factors, such as age, obesity, and joint injury, ML models have revealed novel associations that may contribute to KOA susceptibility. Recent studies have highlighted the role of genetic markers and inflammatory biomarkers in predicting KOA onset, suggesting potential targets for therapeutic intervention [66,70]. Furthermore, ML models can assess the impact of lifestyle factors, such as physical activity levels and dietary habits, on disease risk, providing evidence-based recommendations for modifying risk behaviors [66]. By integrating these diverse data sources, ML offers a comprehensive approach to understanding KOA risk, informing preventive strategies, and optimizing novel therapies.

### 4.3 Current clinical usage and limitations

Predictive modeling using ML is reshaping the landscape of treatment outcome prediction in KOA, particularly in the context of TKA. By analyzing large datasets comprising preoperative clinical information, ML algorithms can forecast postoperative recovery trajectories and potential complications, offering a personalized approach to patient care​ [69]. For instance, models such as random forests and gradient boosting machines have been employed to predict patient-reported outcomes and satisfaction following TKA, outperforming traditional statistical models in accuracy and reliability. These models analyze variables like preoperative pain levels, functional status, and demographic factors to identify patterns that correlate with successful surgical outcomes [67,71].

In addition to improving outcome predictions, ML models are being used to identify patients who are at risk of postoperative complications and dissatisfaction. For example, some models have shown promise in predicting the likelihood of patient dissatisfaction after TKA by identifying factors such as unrealistic expectations, preexisting mental health conditions, and inadequate pain management​ [72]. By identifying at-risk patients preoperatively, clinicians can implement tailored interventions to mitigate these risks, ultimately enhancing patient satisfaction and surgical success rates. The use of ML in this context not only optimizes patient outcomes but also improves resource allocation and reduces healthcare costs​ [73,74].

Despite these benefits, the implementation of predictive modeling in clinical practice faces several hurdles. The accuracy and reliability of these models depend on the availability of high-quality data, including comprehensive preoperative, intraoperative, and postoperative information [67,75]. Additionally, the integration of these models into clinical workflows requires interoperability with existing electronic health record systems and clinician training to interpret and act on model predictions effectively. As with other applications of ML trained on large datasets, concerns related to algorithmic bias and transparency must be addressed to ensure equitable and fair treatment recommendations​.

## 5 Current targets for novel cell based therapies

### 5.1 Pain modulation

Pain induced by KOA is a leading cause of disability and the most frequent reason patients seek medical care [4,63,76]. With nearly a third of adults in the United States at risk of developing KOA in their lifetime due to obesity, pain management of KOA is an important target for novel therapies [63]. The main goal of traditional therapies is the attenuation of pain to promote functionality and improve quality of life [14,76]. Unfortunately, most accepted therapies tend to be short-lived with unfavorable side effects. Oral NSAIDS and opioids have been found to significantly alleviate temporary pain; however, they are associated with adverse effects in the gastrointestinal and central nervous systems [77,78]. To make the management of pain even more complex, radiographic findings regarding the structural pathology of the knee joint do not provide a good indication of the severity of pain in patients [4,76]. Although the etiology of pain in KOA is multifactorial, it is thought that pain results from loss of cartilage that exposes vascular periosteum to high friction movements that stimulate nociceptive nerve endings and prevent adequate absorption of mechanical shock [20,76]. Furthermore, the expression of cytokines in KOA tissue damage may interact with nociceptors within the joint and produce or enhance pain [79]. Ultimately, the goal is to develop an accessible, affordable treatment that can alleviate these symptoms, thereby encouraging an active and healthy lifestyle. In patients with KOA, stem cells have shown promising potential as a safe therapeutic that promotes pain reduction through a variety of mechanisms [80,81].

### 5.2 Cartilage regeneration

The main limitation of current treatments is an inability to regenerate lost cartilage [15]. Because of this, cartilage regeneration is an ideal target for novel stem cell therapies. Despite the fact knee cartilage is strong and durable, it lacks intrinsic self-healing properties because of its avascular and aneural anatomy. This limited access to nutrients within blood restricts the capacity of chondrocytes to replicate efficiently [62,82,83]. Stem cells show encouraging preclinical and clinical results, demonstrating an ability to delay cartilage loss and even increase cartilage volume after substantial joint damage, possibly by amplifying local growth factors and immunomodulatory properties [84].

### 5.3 Inflammation control

Another valuable property of stem cells is their ability to reduce inflammation. Chronic inflammation plays a large role in the pathophysiology of KOA that leads to cartilaginous degeneration and excessive pain [14,85]. When chondrocytes detect abnormal mechanical loading and destructive proteins from the subchondral bone, they are thought to react by releasing cytokines, chemokines, alarmins, damage-associated molecular patterns (DAMPs), and adipokines, which are paracrine factors that travel into the synovium and act on synoviocytes to cause an inflammatory response that is aided by synovial macrophages and fibroblasts. Together with intense inflammation, these catabolic molecules cause a cycle of cartilage degeneration [86]. Stem cells pose a great solution to reduce inflammation, as their immunosuppressive characteristics include the downregulation of T cells, cytokines, and other factors that induce inflammation and upregulate catabolic activity in chondrocytes [63,[87], [88], [89]].

## 6 Stem cells and knee osteoarthritis

All stem cells have two defining qualities: extended self-renewal capabilities and the potential to asymmetrically differentiate into distinct cell types [90]. These characteristics make stem cells extremely advantageous in clinical applications, which include regeneration of damaged or destroyed tissues, modulation of the immune response via interactions with leukocytes, and inflammation regulation through paracrine signaling. Studies have shown that as the severity of OA in the knee progresses, the concentration of MSCs in the synovial fluid drastically increases. These cells mobilize to injured sites, but it is suspected that because of a limited number of MSCs reserved in the synovium, they are unable to hinder the advancement of OA [91]. Consequently, it is no surprise that stem cells are thought to be a viable therapeutic capable of improving the clinical management of KOA. This section aims to highlight significant discoveries in the field of KOA that pertain to the utilization of a diverse range of stem cell varieties, such as MSCs, embryonic stem cells (ESCs), and induced pluripotent stem cells (iPSCs).

A database query was conducted on PubMed using the search terms: “stem cell” AND “knee” AND “osteoarthritis” with the following filters applied: “Clinical Trial” and “Randomized Controlled Trial.” Results were carefully reviewed to generate a comprehensive and up-to-date overview of the current therapeutic utilities of stem cells in KOA treatment.

### 6.1 Mesenchymal stem cells (MSCs)

The scientific inquiry into the impact of stem cells on the advancement of KOA predominantly focuses on the intra-articular injection of MSCs. MSCs are adult cells of multipotent nature that can be extracted from several sources, such as bone marrow, adipose tissue, Wharton's jelly, umbilical cord blood, and peripheral blood. These cells can differentiate into several mesoderm-derived tissues, such as adipose tissue, bone, cartilage, muscle, and other connective tissues [[92], [93], [94], [95]]. The leading motives behind the use of MSCs in KOA clinical trials are their known capacity for immunomodulation, ability to mitigate inflammation, and regenerative qualities that reduce cell apoptosis and promote replacement of lost cells [11,96]. Furthermore, studies have demonstrated that by utilizing autologous MSCs, the hazards of transplant rejection can be avoided, thereby minimizing potential complications and ensuring patient safety [81]. By analyzing eight RCTs, Cao et al. concluded that employing intra-articular injection of MSCs as the sole intervention, regardless of the stem cell source, could significantly reduce pain and improve function in patients with unoperated KOA [97]. Nonetheless, the analgesic effect of MSC therapy remains challenged, such as in the meta-analysis by Sadeghirad et al. which established moderate certainty evidence for little to no improvement in pain or physical function among KOA patients with chronic pain provided by intra-articular MSC injections [98]. While the exact pathophysiological mechanisms underlying the therapeutic effects of MSCs on KOA remain elusive, published literature suggests that these cells possess favorable properties.

### 6.2 Bone marrow-derived MSCs

Current research endeavors primarily center on exploring the impact of MSCs derived from various sources on mitigating pain reported by patients, enhancing knee functionality, and augmenting cartilage volume. Bone marrow-derived mesenchymal stem cells (BM-MSCs) are the most studied MSCs. BM-MSCs are harvested via bone aspirates and promote repair of damaged cartilage through paracrine signaling cascades that involve the secretion of compounds such as bone morphogenetic protein-2 (BMP2) and Insulin-like growth factor-1 (IGF1). These paracrine factors stimulate cellular regeneration and bone formation through stimulating differentiation of progenitor cells in numerous tissues and decreasing OA inflammation and the immune response [13]. The BM-MSCs mechanism of healing most likely involves migration towards the site of injured tissue, where they differentiate into chondrocytes or osteoblasts and commence the mending process by generating elements of the extracellular matrix and repairing lost bone [[99], [100], [101]]. Doyle et al. compared 14 studies focusing on intra-articular injection of BM-MSCs and concluded that BM-MSCs are a safe therapy that improves pain, function, and delays cartilage degradation [13]. A RCT of 24 KOA patients showed improvement in the Western Ontario and McMaster Universities Osteoarthritis Index (WOMAC) and Knee Injury and Osteoarthritis Outcome Score (KOOS) after intra-articular injection of BM-MSCs compared to the saline control group, supporting the pain relief and disease-slowing effect of BM-MSCs [102]. Similarly, Gupta et al. conducted an RCT of 146 patients with grade 2 and 3 OA, demonstrating significant improvements in the WOMAC total score as well as pain, stiffness, and physical function subscores, with preservation of cartilage volume among the intra-articular BM-MSC cohort [103]. Several research articles showed that BM-MSCs were able to increase both articular cartilage and meniscus thickness on radiographic images such as MRIs [[104], [105], [106]]. Wong et al. examined the impact of autologous and allogeneic BM-MSCs after microfracture and open-wedge HTO and found that both types of BM-MSCs injected with HA significantly improved patients’ pain, according to International Knee Documentation Committee (IKDC) scores, and cartilage thickness at two years post-surgery, based on Magnetic Resonance Observation of Cartilage Repair Tissue (MOCARTT), compared to HA injections alone [107]. A similar study, injecting BM-MSCs after arthroscopic debridement, found that autologous and allogeneic BM-MSCs improved cartilage thickness compared to controls [108]. One meta-analysis compared 14 random clinical trials evaluating the use of autologous MSCs and noted that autologous BM-MSCs improved patient-reported pain and painless walking distance compared to saline, significantly improved Knee Society Score (KSS) compared to exercise alone, and significantly reduced pain compared to CS injections after one year [81,[109], [110], [111]]. These findings illustrate the benefits of BM-MSCs compared to current traditional therapies and how they can be augmented alongside other therapies.

Despite the indications of favorable clinical potential of BM-MSCs, they do have several disadvantages. For instance, BM-MSCs may not be an optimal option for older patients who wish to have autologous stem cell injections. As people age, the quantity of BM-MSCs decreases, and cells lose a portion of their regenerative potential [112,113]. In addition, harvesting BM-MSCs is a complex process that extracts a relatively small quantity of stem cells (0.001-0.01% of the removed bone marrow) that may occur with undesirable pain and morbidity [114,115]. Consequently, scientists have focused on exploring other MSC populations that may be more advantageous.

### 6.3 Adipose-derived MSCs

**Fig. 3:**
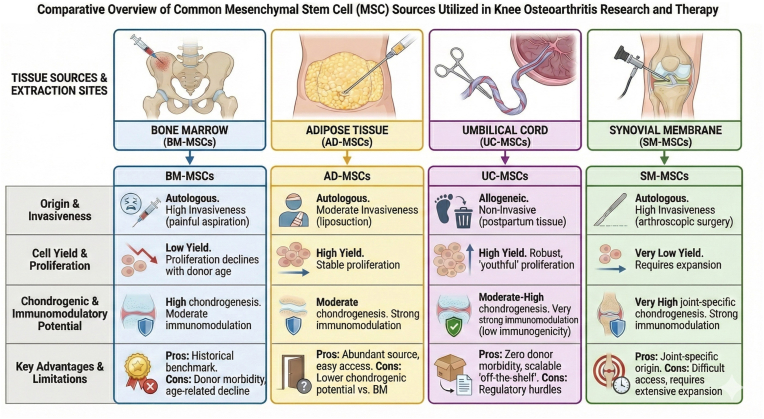
**Comparative Overview of Mesenchymal Stem Cell (MSC) Sources for Knee Osteoarthritis Research and Therapy.** This table presents a side-by-side comparison of the most common tissue sources for MSCs utilized in knee osteoarthritis (KOA) regenerative medicine: Bone Marrow (BM-MSCs), Adipose Tissue (AD-MSCs), Umbilical Cord (UC-MSCs), and Synovial Membrane (SM-MSCs). It delineates key differences regarding harvest invasiveness, initial cell yield, age-related proliferation capacity (“stemness”), and chondrogenic versus immunomodulatory potential. While BM-MSCs serve as the historical benchmark, alternative sources like AD-MSCs and UC-MSCs are increasingly utilized to overcome limitations related to donor morbidity and cell senescence. Synovial MSCs are highlighted for their superior joint-specific differentiation potential despite harvest challenges [40,128].

Compared to BM-MSCs, adipose-derived mesenchymal stem cells (AD-MSCs) are more readily obtainable, produce a higher number of stem cells per unit of tissue removed, exhibit superior regenerative abilities, and possess better properties for stimulating the growth of blood vessels and bones *in vivo* [113]. Although the biological characteristics and capabilities of AD-MSCs vary depending on their location, it is auspicious that these cells can be procured noninvasively from various sources [[116], [117], [118], [119]]. AD-MSCs are thought to work in a fashion very similar to BM-MSCs. They tolerate the hypoxic condition within articular cartilage, demonstrate the potency to differentiate into chondrocytes, and alleviate symptoms of KOA via paracrine signaling [120,121]. Numerous studies have forecasted their potential, emphasizing their capacity to alleviate pain, improve patient physical function, and positively affect the volume of cartilage within the knee joint (Fig. 3) [122,123]. Freitag et al. concluded that in patients with symptomatic KOA, intra-articular injection of autologous AD-MSCs was a safe treatment that significantly improved pain and function 12-months after injection and appeared to have the potential to modify disease progression based on MRI images [121]. In addition, in a 2014 RCT, patients receiving autologous AD-MSCs reported decreased pain two years after intra-articular injection compared to controls [124]. A more recent RCT published in 2025 included 20 patients with idiopathic KOA, half of whom received an intra-articular AD-MSC injection and exhibited significant pain reduction and an increase in cartilage thickness compared to the saline group, further delineating the regenerative potential of AD-MSCs in certain knee compartments [125]. Not only did AD-MSC show cartilage regeneration potential in patients with advanced KOA, but it also led to a significant reduction of cartilage lesions among patients with early KOA, in addition to continuous improvements in WOMAC pain, stiffness, and function scores [126]. Zhao et al. gathered data from 43 RCTs using a network meta-analysis and found that compared to saline (control), HA, and BM-MSCs, AD-MSCs were the most effective in reducing pain, and the clinical efficacy of AD-MSCs was better than that of BM-MSCs [120]. AD-MSCs have even been found to provide benefit when used with other novel therapies, such as PRP, a treatment presumed to promote musculoskeletal tissue repair through supraphysiologic quantities of growth factors that act as regenerative stimuli [127]. In fact, Koh et al. investigated the effect of intra-articular injection of PRP with autologous AD-MSCs compared to PRP alone after knee arthroscopy and HTO surgery and found that PRP with MSCs improved pain, function, and resulted in better fibrocartilage coverage after two years [124]. These studies have demonstrated that AD-MSCs exhibit many of the favorable traits of BM-MSCs, along with a host of superior properties, rendering adipose tissue an ideal source of MSCs for treating KOA.

### 6.4 Perinatal MSCs

Moreover, the non-invasive isolation, anti-inflammatory capability, and high proliferation potential of human placenta-derived MSCs (hP-MSCs) and human umbilical cord MSCs (hUC-MSCs) made them another investigative target for KOA treatment besides BM-MSCs and AD-MSCs [129,130]. Holiuk et al. conducted a non-randomized prospective study of 26 patients with stage II-III KOA, applying three intra-articular injections of hP-MSCs to the experimental group, which showed significant improvements in WOMAC and VAS scores compared to the control group. Interestingly, the authors also noted a significant decrease in interleukin-2 levels in the hP-MSC group, indicating an anti-inflammatory effect [129]. On the other hand, Emadedin et al. and Pico et al. applied hUC-MSCs in treating KOA, with results all agreeing on the pain reduction effect [111]. Furthermore, Wang et al. used hUC-MSC-derived exosomes, which could reduce immune rejection and carcinogenicity while being modifiable for enhanced targeting ability, therapeutic efficacy, and delivery capacity. However, the advantages of exosomes over whole stem cell treatments need to be further explored, along with the benefits and harms, when determining the superiority of one treatment over the other [130].

### 6.5 Cell retention and biomaterial support

Although AD-MSCs can overcome several drawbacks of BM-MSCs, there remain additional limitations. For example, to utilize MSCs as a clinical therapeutic, there must be a sufficient quantity of useable cells. Therefore, it is often required that these cells be expanded *ex vivo*. This is a complex process that may reduce clinical efficacy and increase the risk of malignant mutations [131,132]. Moreover, in KOA, the continuous circulation of synovial fluid that occurs when MSCs are injected within the joint prevents MSCs from localizing to injured sites [13]. As a result, many experts believe MSCs primarily alleviate symptoms of KOA via paracrine signaling rather than differentiation into chondrocytes. To improve stem cell retention, biomaterials that may aid MSCs in cartilage regeneration have been developed. Various scaffolds, including sponges/foams, fibrous matrices, and layered structures, have already shown significant potential in clinical studies, improving the efficacy of MSCs by providing a supportive and protective environment that enhances their regenerative potential [63]. Sakamoto et al. reported a successful increase in AD-MSCs’ survival in the intra-articular environment by adhering to atelocollagen microspheres [133]. Paradiso et al. combined 3D biomimetic scaffolds with synovial fluid-derived MSCs, which promoted immunosuppressive marker expression and further enhanced the immune tuning capacity of MSC therapy [134]. However, whether scaffold-implanted MSCs can challenge the well-established acellular scaffolds with concentrated bone marrow aspirate therapy for cartilage repair needs to be answered by stronger future evidence [135].

### 6.6 Pluripotent stem cells

The limitations of multipotency with MSCs may be overcome with the use of pluripotent ESCs. However, there are numerous ethical obstacles surrounding ESC research. Namely, projects with ESCs are subject to the ethical considerations of human experimentation. Cultivation of ESCs also causes the destruction of an embryo, which is a focal point of discussion. Another concern is the false advertising of a treatment's capabilities, as Stem Cell Treatment guidelines can vary from different countries. To ensure that this practice is properly regulated, Gopalan et al. recommended the adoption of guidelines from the International Society for Stem Cell Research, which included secular and universal moral guidelines to shape research and practice with ESC [136].

One method to utilize the pluripotent potential of ESCs without ethical concerns is iPSCs. iPSCs are generated by exposing adult cells to specific transcription factors that reprogram cells back to a pluripotent state similar to that of ESCs [137]. The reprogramming process is yet to be fully understood, but with the ability to regenerate into any cell type within the human body, iPSCs should be applicable towards regenerative medicine. Kamaraj et al. performed a systematic literature review including eight studies that examined the ability of human iPSCs to regenerate cartilage in vitro and repair cartilage defects in the knees of animals. Since most studies found that human iPSCs could regenerate high-quality cartilage that allowed for cartilage repair *in vivo* with no tumorigenicity observed, Kamaraj and colleagues concluded that human iPSCs have the potential to serve as a promising therapy in improving cartilage regeneration in knees [138]. Even though animal models show good results, we await the application of iPSCs in clinical trials and must consider the potential adverse side effects of iPSCs, such as malignant mutations, that may result from their use [139,140]. By further understanding the biological characteristics of iPSCs, their qualities may be used to overcome the limitations of currently accepted therapies for KOA.

Although Stem Cell Research offers novel treatments for degenerative conditions such as KOA, human stem cell research (hSCR) raises several ethical concerns [141]. hSCR involves the derivation of pluripotent stem cell lines from oocytes and embryos, but this process is replete with ongoing disputes with respect to the onset of human personhood and reproduction. Reprogramming somatic cells to produce induced iPSCs does help mitigate some of these ethical concerns. However, additional concerns persist regarding informed consent for tissue donation, the conduct and oversight of clinical trials, and the equitable regulation of hSCR across institutions [141].

### 6.7 Ethical considerations

Ethical dilemmas can emerge at any stage of hSCR. Donating biological materials requires clear, voluntary, informed consent. Research involving human embryonic stem cells (hESCs) is particularly controversial due to the destruction or creation of embryos for research purposes, potential exploitation of oocyte donors, medical risks of oocyte retrieval, and the need to safeguard women's reproductive interests. Furthermore, the use of stem cell lines derived at other institutions raises concerns about conflicting legal or ethical standards, and stem cell clinical trials raise concerns about the risks or benefits of the experimental intervention [141].

iPSCs are obtained from the inner cell mass of a 5- to 7-day-old blastocyst, a process that entails embryo destruction and thus provokes fundamental ethical concerns about when human life begins. Critics of hSCR argue that embryos possess the same moral status as living humans, making such research equivalent to taking human life [141,142]. Others maintain that moral status develops later in embryonic development, while many adopt a middle-ground position–accepting research on embryos if it is scientifically justified, ethically reviewed, and performed with informed consent [141,143].

Although hSCR holds immense potential to advance regenerative medicine and improve outcomes in KOA, the use of stem cells raises concern about complex ethical and societal issues. These concerns must be carefully navigated to ensure that research proceeds in a transparent, responsible, and ethically sound manner aimed solely at improving the clinical care of patients.

## 7 Limitations and future directions

Stem cell research aimed at improving KOA has numerous limitations. Firstly, more double-blinded RCTs with larger cohorts are needed to further elucidate the role of mesenchymal stem cells in KOA treatment. As of now, it remains difficult to draw strong conclusions and generalizations regarding stem cell-based therapies in KOA due to small cohort sizes, short follow-up durations, and a lack of standardized protocols in the administration of stem cells, reducing the statistical power of studies. Furthermore, exclusion biases in the selection of patients based on age, previous knee joint trauma, comorbidities, anatomical or functional impairments, and psychological factors may further reduce the generalizability of published studies [105,110,123].

Several different survey modalities to assess joint pain and quality of life during the administration of stem cell therapies were used across studies, including the WOMAC, visual analog scale (VAS), intermittent and constant osteoarthritis pain (ICOAP), numerical pain rating scale (NRS), and short form 36 (SF-36), among other surveys. While these surveys provide a way for clinicians to assess pain and functionality of the joints over the duration of treatment, they come with their own limitations. First, inconsistency between surveys makes it difficult to draw conclusions across studies. Additionally, the questionnaires rely on the subjective evaluation of the patient's physical pain, which may be influenced by a variety of factors, including pain tolerance and emotional states. Furthermore, the use of imaging modalities to support trends in pain and functionality does not always show statistical significance as compared to baseline or placebo.

Emerging applications of ML may help overcome some of these limitations by providing standardized, objective, and data-driven approaches to evaluating KOA progression and treatment response. ML algorithms can integrate multimodal data such as clinical records, imaging studies, and biochemical markers to identify predictive biomarkers, automate radiographic grading, and model patient outcomes with greater accuracy. These tools can also enhance trial design by identifying ideal candidate subpopulations and optimizing treatment parameters, which may ultimately improve the reproducibility and clinical applicability of stem cell studies.

Moving forward, it is vital to ensure that the selection of stem cell therapies be held to a criterion that allows for reproducibility and comparison across studies. To this end, the Mesenchymal and Tissue Stem Cell Committee of the International Society for Cellular Therapy (ISCT) has provided characterization methods for mesenchymal stem cells. Additionally, protocols on the number and frequency of stem cells injected should be further established with dose-response relationship studies.

## 8 Conclusion

KOA is a degenerative condition that damages the knee joint's structural integrity, causing significant disability worldwide. It leads to chronic pain, functional limitations, and costly treatments, thus adversely affecting patients' quality of life. Although current treatments and surgeries are critical for helping patients with KOA, stem cells show promise as a non-surgical therapy that could potentially halt and even reverse the condition's progression. Simultaneously, ML offers new opportunities to enhance the management of KOA by improving diagnostic accuracy, predicting treatment outcomes, and personalizing therapeutic strategies. However, to evaluate the use of different stem cell populations in clinical applications of KOA and integrate ML-based tools into clinical practice, clinicians and researchers need to develop standardized methods that reduce heterogeneity between studies. In addition to more randomized clinical studies with larger cohorts, optimal doses, sources, and frequency of stem cells need to be established to determine whether they represent a viable solution to the current challenges of KOA.

## Author contributions

**Arthur W. Cowman:** conceptualization, formal analysis, methodology, writing – original draft (lead), writing – review and editing.

**Jordan N. Tang:** conceptualization, formal analysis, methodology, writing – original draft, writing – review and editing.

**Jeffrey Deng:** conceptualization, formal analysis, methodology, writing – original draft, writing – review and editing.

**Ammar Abu-Halawa:** conceptualization, formal analysis, methodology, writing – original draft, writing – review and editing.

**Haiyue Jin:** conceptualization, formal analysis, methodology, writing – original draft, writing – review and editing.

**Christopher Z. Liu**: formal analysis, methodology, writing – review and editing.

**Ryan Hoang:** conceptualization, formal analysis, methodology, writing – original draft, writing – review and editing.

**Walter Cowman:** conceptualization, formal analysis, methodology, writing – review and editing.

**Michael S. Kim:** conceptualization, formal analysis, methodology, writing – review and editing.

**Sultan Baz:** conceptualization, formal analysis, methodology, writing – review and editing.

**MacKinnly T. Knoerzer:** methodology, writing – review and editing.

**Amir-Ala Mahmoud:** conceptualization, formal analysis, methodology, writing – review and editing.

**Amir-Ali Mahmoud:** formal analysis, methodology, writing – review and editing.

**Miles D. Melamed:** formal analysis, methodology, writing – review and editing.

**Hong-Wen Deng:** conceptualization (lead), formal analysis (lead), methodology (lead), writing – original draft, writing – review and editing (lead).

**Table:** Glossary

Abbreviation	Term
OA	Osteoarthritis
KOA	Knee osteoarthritis
TKA	Total knee arthroplasty
NSAIDs	Nonsteroidal anti-inflammatory drugs
HA	Hyaluronic acid
MSCs	Mesenchymal stem cells
ML	Machine learning
MMPs	Metalloproteinases
ADAMTS	A disintegrin and metalloproteinase with thrombospondin motifs
COX	Cyclooxygenase enzymes
OARSI	Osteoarthritis Research Society International
CS	Corticosteroid
RCTs	Randomized clinical trials
PRP	Platelet-Rich Plasma
LRPRP	Leukocyte-Rich Platelet-Rich Plasma
LPPRP	Leukocyte-Poor Platelet-Rich Plasma
HTO	High tibial osteotomy
CNN	Convolutional neural networks
KL	Kellgren-Lawrence
DAMPs	Damage-Associated Molecular Patterns
ESCs	Embryonic stem cells
iPSCs	Induced pluripotent stem cells
BM-MSCs	Bone marrow-derived mesenchymal stem cells
BMP2	Bone morphogenetic protein-2
IGF1	Insulin-like growth factor-1
WOMAC	Western Ontario and McMaster Universities Osteoarthritis Index
IKDC	International Knee Documentation Committee
MOCARTT	Magnetic Resonance Observation of Cartilage Repair Tissue
KSS	Knee Society Score
AD-MSCs	Adipose-Derived Mesenchymal Stem Cells
hP-MSCs	Human Placenta-Derived Mesenchymal Stem Cells
hUC-MSCs	Human Umbilical Cord Mesenchymal Stem Cells
hSCR	Human Stem Cell Research
hESCs	Human Embryonic Stem Cells
VAS	Visual Analog Scale
ICOAP	Intermittent and Constant Osteoarthritis Pain
NRS	Numeric Pain Rating Scale
ISCT	International Society for Cellular Therapy
SF-36	Short-Form 36
UKA	Unicompartmental Knee Arthroplasty
SM-MSCs	Synovial membrane-derived mesenchymal stem cells

## AI disclosure

AI was used in the creation of figure initial drafts with Nano Banana Pro software. All figures were subsequently manually reviewed, edited, and finalized by the authors using Canva and Microsoft PowerPoint to ensure scientific accuracy and consistency with the cited literature. The authors take full responsibility for the accuracy and integrity of all visual content.

## Declaration of competing interest

The authors declare that there is no conflict of interests regarding the publication of this paper.
